# Primary Abdominal Pregnancy in the Mesocolon: A Report of a Rare Case

**DOI:** 10.7759/cureus.91173

**Published:** 2025-08-28

**Authors:** Maria I Sousa, Flávia Ribeiro, Cristiana Moreira, Tânia Barros, José Pedro Santos, Ana Galvão, Sílvia Neves, João André Oliveira, Eugénia Fernandes, Luís Guedes-Martins, Rosa Zulmira Macedo

**Affiliations:** 1 Department of Women and Reproductive Health, Unidade Local de Saúde de Santo António, Porto, PRT; 2 Department of General Surgery, Unidade Local de Saúde de Santo António, Porto, PRT; 3 Department of Gynecology and Obstetrics, Unidade Local de Saúde de Alto Ave, Porto, PRT; 4 Department of Radiology, Unidade Local de Saúde de Santo António, Porto, PRT

**Keywords:** abdominal pain, diagnostic laparoscopy, ectopic pregnancy, hemoperitoneum, pregnancy of unknown location

## Abstract

An abdominal ectopic pregnancy is a rare and potentially life-threatening form of ectopic pregnancy, associated with a high maternal mortality rate.

We report the case of a 25-year-old woman with an abdominal ectopic pregnancy who underwent three surgical interventions, including diagnostic laparoscopy, before definitive diagnosis and treatment. Management involved a multidisciplinary team of gynecologists, general surgeons, and interventional radiologists, using advanced imaging, selective arterial embolization, and laparotomy with removal of the abdominal mass and transverse mesocolon epiploon. This report summarizes our experience with the diagnosis and management of this rare and potentially life-threatening condition.

Despite the diagnostic delay, the pregnancy was identified at an early gestational stage, avoiding more severe complications.

This case illustrates the diagnostic challenges and complexity of managing abdominal ectopic pregnancies, highlighting the importance of careful assessment and individualized treatment. In some cases, a multidisciplinary approach may be essential to ensure optimal outcomes.

## Introduction

Ectopic pregnancy (EP) is defined as the presence of gestational tissue outside the uterine cavity [1,2]. Established risk factors include a history of previous ectopic pregnancy, prior tubal surgery, tubal pathology, sterilization, intrauterine device use, and assisted reproductive techniques such as in vitro fertilization [3]. Abdominal ectopic pregnancy (AEP) is an exceptionally rare form of EP, with an estimated incidence of 1:10,000 to 1:30,000 pregnancies [4,5]. It represents a particularly high-risk entity. Early identification and treatment are often difficult, and the condition is associated with a markedly elevated maternal mortality rate [5,6]. The clinical presentation of AEP is highly variable, and the absence of consistent signs and symptoms makes diagnosis especially challenging. Implantation may occur on the omentum, broad ligament, abdominal organs, or major blood vessels [1], excluding pregnancies that are tubal, ovarian, or secondary to reimplantation following a primary tubal pregnancy [7].

In 1942, Studdiford et al. proposed a classification system distinguishing primary from secondary AEP. Primary AEP is defined by three criteria: (1) normal tubes and ovaries, (2) no evidence of a uteroperitoneal fistula, and (3) pregnancy exclusively related to the peritoneal surface at an early gestational age. If one or more of these criteria are not fulfilled, the case is considered secondary AEP, usually following tubal rupture or expulsion with subsequent reimplantation in the abdominal cavity [8].

Due to its rarity, most published data on AEP comes from case reports or small case series. Management strategies described in the literature include surgical approaches (laparotomy or laparoscopy), medical treatment (such as intramuscular or intralesional methotrexate and/or intracardiac potassium chloride), or combined medical-surgical approaches [7].

We report a case of primary AEP located in the mesocolon, highlighting the prolonged diagnostic process, which required multiple interventions, and the therapeutic challenges associated with this rare yet clinically significant condition.

## Case presentation

We present the case of a healthy 25-year-old white woman, G2P0 (previous medically interrupted pregnancy for fetal malformation). In September 2024, the patient presented at our emergency department at six weeks and two days of amenorrhea, complaining of suprapubic pain with a three-day evolution. A serum beta-human chorionic gonadotropin (β-hCG) value of 4,198 IU/L had been obtained at another institution the previous day. The patient also reported a single episode of light vaginal bleeding two weeks prior. Upon examination, she was hemodynamically stable, afebrile, and presented with lower abdominal quadrant pain, without signs of peritoneal irritation.

Gynecological examination was unremarkable. A pelvic ultrasound revealed an endometrial thickening of 15 mm, a gestational sac-like echo measuring 8 mm, and a right adnexal mass measuring 35×35×34 mm, consistent with a hemorrhagic cyst. Serum β-hCG was 5108 IU/L. As her symptoms resolved, she was scheduled for reevaluation in 48 hours. On return, the patient reported mild hypogastric intermittent pain, worsened by body movements. Ultrasound revealed a moderate amount of free fluid in the pouch of Douglas, with no increase in the size of the intrauterine gestational sac-like structure. A serum β-hCG of 7,998 IU/L and hemoglobin level of 11.7 g/dL were obtained, showing a suboptimal increase in β-hCG levels. Table 1 presents the patient’s clinical presentation, β-hCG and hemoglobin levels, and the corresponding clinical management over time. A pregnancy of unknown location was assumed, and diagnostic laparoscopy was performed. During the procedure, moderate hemoperitoneum was drained, and pelvic organs appeared macroscopically normal, except for the right ovary, which showed evidence of active hemorrhage. Rupture of a hemorrhagic corpus luteum was suspected, and hemostasis was achieved. The drained material was sent for histopathological evaluation and showed no trophoblastic tissue.

**Figure 1:**
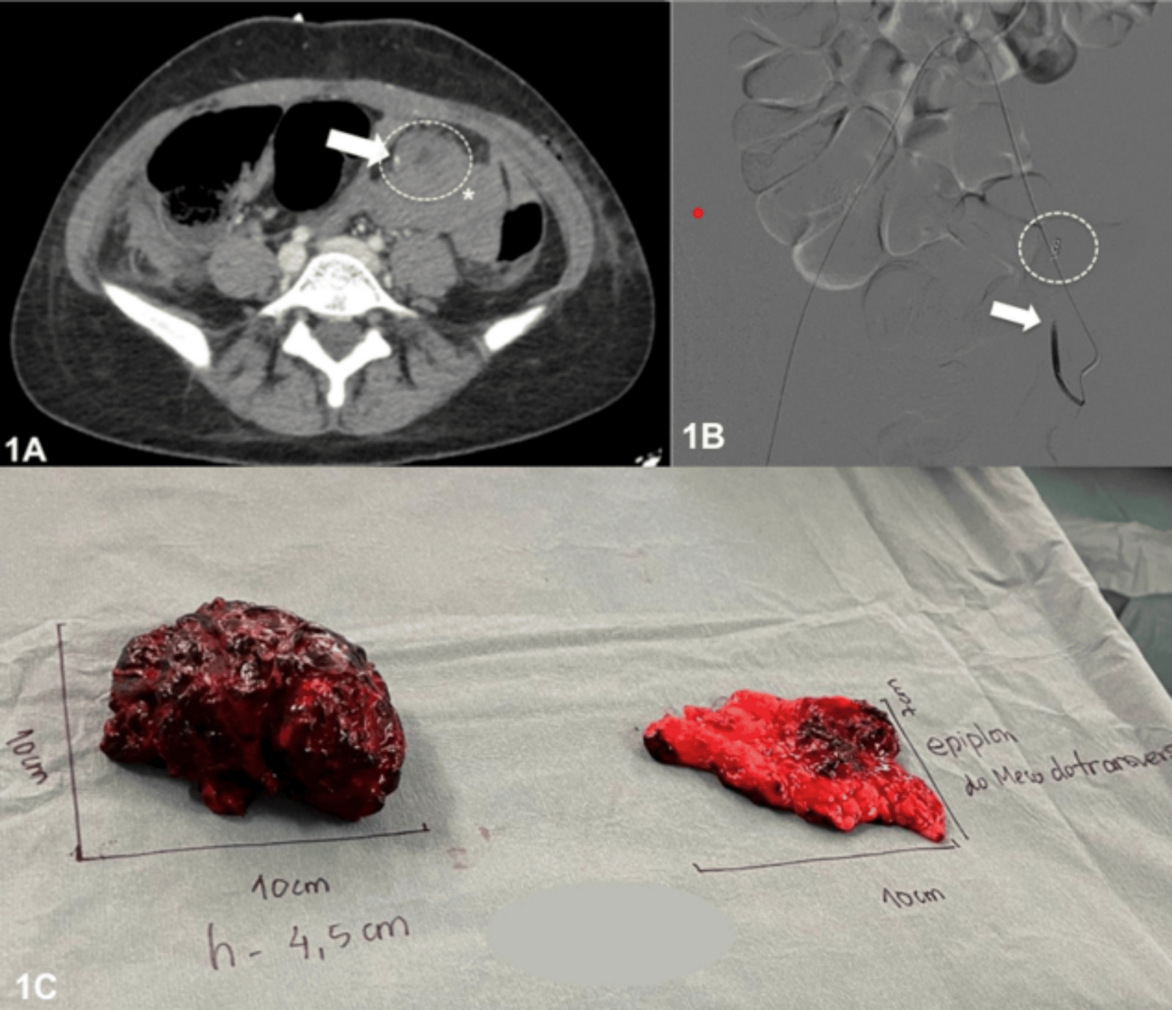
Radiological and surgical findings in the abdominal ectopic pregnancy Axial computed tomography image showing moderate hemoperitoneum (white asterisk) and a small arterial blush (white arrow) of uncertain origin. The blush measures approximately 3 mm and becomes nearly isodense in the delayed phase, possibly representing a low-flow bleeding focus in an interloop location. A possible abdominal ectopic pregnancy is retrospectively identified (dashed circle). (B) Digital subtraction angiography after coil embolization of the distal inferior epigastric artery (dashed circle) and particle embolization of the proximal segment and an anomalous intraperitoneal branch (white arrow). (C) Surgical specimen: organized blood clots (left) and abdominal mass corresponding to the ectopic pregnancy attached to the epiploon of the transverse mesocolon (right), excised via laparotomy.

On postoperative day 1, the patient developed hypotension and signs of peritoneal irritation. A second diagnostic laparoscopy revealed a large hemoperitoneum and ongoing right ovarian bleeding, leading to a right salpingo-oophorectomy. Two units of packed red blood cells were transfused intraoperatively. Despite this, her hemodynamic status worsened, and hemoglobin levels continued to decline. The day after, an abdominal-pelvic computed tomography (CT) scan showed moderate hemoperitoneum with a small arterial blush in the interloop region of the left flank, suspected as the bleeding source (Figure 1A). On the same day, angiography, with selective catheterization of the left iliac artery via a crossover approach and the superior epigastric artery, identified intraperitoneal branches feeding the hemorrhagic focus. Embolization of an anomalous intraperitoneal branch of the left inferior epigastric artery was performed using polyvinyl alcohol particles followed by microcoils, achieving successful vascular occlusion (Figure 1B). At that time, this bleeding was thought to have possibly resulted from trocar placement in the left iliac fossa during the previous laparoscopic procedure.

The following day, β-hCG levels showed a suboptimal decrease, which could indicate that residual trophoblastic tissue remained in the peritoneal cavity. This interpretation is supported by the continued decline in hemoglobin levels despite transfusional support (Table 1), suggesting ongoing blood loss. Meanwhile, histopathological evaluation of the previously removed adnexal tissue showed no evidence of ectopic pregnancy.

**Table 1:** Patient’s clinical presentation, serum β-hCG trend, and timeline of events CT: computed tomography; RR: reference range; β-hCG: beta-human chorionic gonadotropin

Number of days after initial presentation	Clinical presentation	Serum β-hCG level (UI/L) (RR: ≤ 1 U/L)	Hemoglobin level (g/dL) (RR: 12.0-15.0 g/dL)	Approach/Event
1	Suprapubic pain with 3 days of evolution	5108	12.9	Re-evaluation at 48h
3	Light intermittent hypogastric pain	7998	11.7	Laparoscopy with hemoperitoneum drainage
4	Hypotensive profile with abdominal pain and signs of peritoneal irritation	NA	7.2 >> 8.9 (after transfusion)	Transfusional support. Laparoscopy with hemoperitoneum drainage (1500 mL) and right adnexectomy
5	Hypotension	2846	7.4	Transfusional support. Abdominal and pelvic CT; angiography and left inferior epigastric artery embolization
6	Clinical improvement	2308 - 1 day after arterial embolization	7.2	Clinical and laboratory follow-up
7	Abdominal pain	1900	7.6	Transfusional support. Diagnostic laparotomy with a mesenteric ectopic pregnancy, total removal.
8	Pain improvement	1116 - 1 day after surgical removal	9.4	Clinical and laboratory follow-up
14 (Hospital discharge)	Asymptomatic	75.8	9.7	Medical appointment scheduled for reevaluation one week later
31	Asymptomatic	<2	10.5	Medical appointment scheduled six months later for preconception counseling

Given the persistent clinical deterioration and inconclusive prior interventions, an exploratory laparotomy was then performed through a multidisciplinary approach involving both general surgeons and gynecologists. Considering the strong suspicion of an AEP, this decision reflected the need for definitive management after standard diagnostic and minimally invasive approaches had failed to identify and control the source of bleeding. During exploratory laparotomy, a moderate hemoperitoneum was identified. A firm, irregular 10 cm mass on the epiploon of the transverse colon, surrounded by blood clots and adherent to the transverse mesocolon, was excised. Histopathology confirmed an AEP, showing adipose tissue with adherent trophoblastic cells and chorionic villi within the clots, while the 10 cm mass corresponded to organized blood clots (Figure 1C). The patient’s β-hCG levels showed a significant decrease the following day, and hemoglobin levels and the patient’s clinical condition improved progressively during the hospital stay, supporting the resolution of the ectopic pregnancy. She was discharged home in a clinically stable condition on day 14 after admission. Serum β-hCG levels were measured weekly and became negative approximately one month after the initial diagnosis, without any further intervention (Table 1).

## Discussion

Herein, we report a case of abdominal pregnancy presenting with moderate hemoperitoneum at an early gestational stage. Based on Studdiford’s criteria, this case qualifies as a primary AEP [8,9], likely resulting from fertilization of the oocyte within the abdominal cavity [1,2].

A high index of suspicion is needed to diagnose AEP [10]. Despite focused antenatal care and advanced imaging modalities, only 20-40% of cases are diagnosed preoperatively [1,3]. Since typical signs and symptoms of tubal ectopic pregnancy may be absent in cases of primary AEP [9,11], most abdominal pregnancies are diagnosed only after complications have occurred. In rare cases, they may remain asymptomatic, and diagnosis may be delayed until advanced gestational age [1,3].

In this case, the onset of mild symptoms during the first trimester in a woman with a history of a medically interrupted pregnancy prompted early investigation. At initial presentation, the pregnancy was classified as a pregnancy of unknown location, guiding the first steps of diagnosis and management [2,4]. The primary differential diagnosis was an adnexal ectopic pregnancy. During the initial diagnostic laparoscopy, the bleeding ovary was interpreted as a corpus luteum, with no evidence of pregnancy, leading to consideration of a possible miscarriage of an ectopic pregnancy. To maintain a minimally invasive approach, a second laparoscopy was subsequently performed, initially interpreted as a right ovarian ectopic pregnancy. Careful correlation of clinical presentation, imaging findings, and biochemical markers proved essential in distinguishing among these possibilities and guiding management [3,5]. In retrospect, performing a laparotomy with comprehensive exploration of the abdominopelvic cavity might have enabled an earlier diagnosis and potentially avoided additional interventions [5,6].

Anemia refractory to transfusional support and suboptimal serum β-hCG decrease after surgery were important clues prompting further investigation, ultimately leading to the correct diagnosis of primary AEP [12]. In general, β-hCG levels are expected to decline by more than 50% within the first 2 days after surgery and continue to decrease progressively, reaching undetectable values within 2-3 weeks in most cases [13,14]. In our patient, the suboptimally declining β-hCG following adnexectomy may reflect corpus luteum removal rather than definitive resolution of the ectopic pregnancy. A similar pattern after embolization may indicate partial treatment, with interruption of vascularization to the ectopic site [14]. Ectopic trophoblastic implantation can recruit abnormal vessels, as observed in imaging studies of our case [14]. Serial β-hCG and hemoglobin monitoring provided critical insight into treatment effectiveness and hemodynamic stability [13,14].

Pelvic ultrasound often fails to diagnose AEP [9,11], and although advanced imaging modalities, such as CT and MRI, can be helpful for diagnosis and surgical planning [9,15], they may also miss the condition, as occurred in our case.

Early AEP may sometimes be managed medically when the location is unknown [16]. Although there are no standardized guidelines, treatment is predominantly surgical, either via laparoscopy or laparotomy, with laparoscopy being viable in early gestational stages [15,16]. In our case, laparoscopy for suspected adnexal ectopic pregnancy failed to identify abdominal implantation. When an ectopic pregnancy remains undiagnosed and symptoms persist or worsen, exploratory laparotomy should be promptly considered, performed by an experienced surgeon with appropriate transfusional support [2,6]. This highlights that, in cases of persistent diagnostic uncertainty, an open approach may facilitate timely diagnosis and prevent treatment delays [5,6].

Fessehaye et al. reported a woman initially managed as a missed second-trimester miscarriage who was later diagnosed with AEP via ultrasound [11]. Laparotomy allowed the removal of the fetus and placenta without major hemorrhage, although sigmoid colon injury occurred. This case emphasizes assessing placental attachment to guide whether to remove the placenta or leave it in situ and the importance of safe entry into the abdomen [11].

In our case, laparotomy allowed the visualization of placental implantation on the mesenteric surface and definitive treatment without significant hemorrhage, which may occur due to deep, irregular trophoblastic invasion into surrounding tissue [2,9,11,17].

Preoperative embolization of feeding vessels has been proposed to reduce bleeding risk [14,18], particularly when combined with adjuvant methotrexate therapy [16,18]. In our patient, embolization controlled active hemorrhage from the left inferior epigastric artery. This approach can optimize intraoperative bleeding control in high-risk cases [14,18]. In conditions with a high hemorrhage risk, such as placenta accreta spectrum or implantation in highly vascularized sites (omentum, spleen, liver), some authors recommend leaving the placenta in situ [1,11,18]. The Society of Obstetricians and Gynecologists of Canada emphasizes individualized management for pregnancies of unknown location and non-tubal ectopic pregnancies [2].

Demendi et al. reported a 17-week abdominal pregnancy diagnosed with ultrasound and MRI, followed by angiographic embolization of the supporting artery before laparotomy [18]. The placenta was left in situ due to the high bleeding risk, with methotrexate used postoperatively to accelerate trophoblastic involution. Serum β-hCG guided monitoring.

This case highlights the critical role of multidisciplinary teamwork, with the gynecologist coordinating care, interventional radiology controlling bleeding, and general surgery performing laparotomy and removing ectopic tissue. Close collaboration ensured patient safety, timely interventions, and favorable outcomes [2,14,16].

A systematic review on early abdominal pregnancy reported 25 cases of primary omental ectopic pregnancies [7]. All patients presented with abdominal pain, with diagnosis typically established intraoperatively after excluding tubal pregnancy. Imaging often suggested a ruptured tubal pregnancy due to free fluid and the absence of an intrauterine gestational sac. Notably, 36% required transfusions, with an average blood loss of 1160 mL, highlighting the hemorrhagic potential. Laparoscopy was successful in about one-third of cases, but diagnosis was often delayed. No maternal deaths were reported [7].

Other rare ectopic implantation sites, including rectal and retroperitoneal pregnancies, have also been described. Rectal ectopic pregnancies may be managed with minimally invasive approaches such as local and systemic methotrexate [19], while retroperitoneal ectopic pregnancies can, in selected cases, be managed expectantly during early pregnancy failure [20]. Awareness of these unusual implantation sites is important, as their clinical presentation, diagnostic challenges, and management strategies differ from both tubal and typical abdominal ectopic pregnancies [19,20].

## Conclusions

In conclusion, this case highlights the diagnostic challenges and complexity of managing AEP. In our patient, persistent abdominal pain, suboptimally declining β-hCG levels, and inconclusive ultrasound findings were key clinical indicators that guided further evaluation and ultimately led to the diagnosis. Early recognition, careful assessment, and timely surgical intervention allowed definitive treatment without major maternal complications. This case also underscores the importance of individualized management and illustrates how a multidisciplinary approach can be essential to optimize outcomes in such rare presentations.
